# Presence of Adenovirus Species C in Infiltrating Lymphocytes of Human Sarcoma

**DOI:** 10.1371/journal.pone.0063646

**Published:** 2013-05-06

**Authors:** Karin Kosulin, Franziska Hoffmann, Till Sebastian Clauditz, Waldemar Wilczak, Thomas Dobner

**Affiliations:** 1 Heinrich Pette Institute, Leibniz Institute for Experimental Virology, Department of Molecular Virology, Hamburg, Germany; 2 Department of Pathology, University Medical Center Hamburg-Eppendorf, Hamburg, Germany; Enzo Life Sciences, Inc., United States of America

## Abstract

Human adenoviruses are known to persist in T-lymphocytes of tonsils, adenoids and intestinal tract. The oncogenic potential of different adenovirus types has been widely studied in rodents, in which adenovirus inoculation can induce multiple tumors such as undifferentiated sarcomas, adenocarcinomas and neuroectodermal tumors. However, the oncogenic potential of this virus has never been proven in human subjects. Using a highly sensitive broad-spectrum qRT-PCR, we have screened a set of different human sarcomas including leiomyosarcoma, liposarcoma and gastro intestinal stroma tumors. Primers binding the viral oncogene E1A and the capsid-coding gene Hexon were used to detect the presence of adenovirus DNA in tumor samples. We found that 18% of the tested leiomyosarcomas and 35% of the liposarcomas were positive for the presence of adenovirus DNA, being species C types the most frequently detected adenoviruses. However, only in one sample of the gastro intestinal stroma tumors the virus DNA could be detected. The occurrence of adenovirus in the tumor sections was confirmed by subsequent fluorescence in-situ-hybridization analysis and co-staining with the transcription factor Bcl11b gives evidence for the presence of the virus in infiltrating T-lymphocytes within the tumors. Together these data underline, for the first time, the persistence of adenovirus in T-lymphocytes infiltrated in muscular and fatty tissue tumor samples. If an impaired immune system leads to the viral persistence and reactivation of the virus is involved in additional diseases needs further investigation.

## Introduction

Human Adenoviruses (HADV) are classified into seven species termed A-G, and the number of HAdV types has been increasing over the past years and, owing to very recent additions, currently comprises >60 distinct types [1], [2], [3]. Primary infections caused by HAdVs tend to occur during early childhood, and most commonly affect the gastrointestinal and the respiratory tract. Human adenoviruses display tissue-specific tropism, and their persistence has been shown in adenoids, tonsils, intestine, lung epithelium and central nervous system [4], [5], [6]. A high prevalence of species C types 1, 2 and 5 have been found in the T-Lymphocytes of adenoids and tonsils [4], whereas species E type 4 has been frequently detected in the T-Lymphocytes of the intestinal tract [7]. HAdV has been shown to induce malignant tumors in animals several decades ago. HAdV type 12 belonging to species A is described as a prototype of an oncogenic virus inducing malignancy upon inoculation into newborn hamsters [8], [9] and similar observations have been shown also with other HAdV representatives from species B and D [10], [11]. Injection of the purified virus has been associated with the development of undifferentiated sarcomas, adenocarcinomas, neurogenic and mammary gland tumors in hamsters and rats [8], [10]. However, in contrast to several other tumorigenic viruses [12], [13], [14], data supporting the possible oncogenicity of adenoviruses in humans are scarce. A number of studies reported the absence of HAdV sequences in different malignancies of adult patients including lung, stomach, colon and kidney tumors [15], [16], as well as various types of leukemia [17]. Only few studies reported the presence of HAdV DNA in human cancers. For example, HAdV species C has been detected in small-cell lung carcinoma, but not in other types of lung cancer [18]. A previous study provided evidence for the presence of HAdV DNA in different brain tumors while several specimens from other pediatric tumor entities investigated revealed HAdV-negative findings [6]. Hence, despite the well-established oncogenicity of certain HAdV types in animal models, the possible role of this viral genus in human neoplasia remains elusive. Since HAdV has not been correlated with human neoplasia so far it is regarded to be non-harmful and the use of adenovirus as oncolytic vector became a promising tool in cancer therapy and other diseases. Due to safety reasons and the lack of long-term studies it seems beneficial studying the oncogenicity of HAdV for humans.

The adenoviral tumorigenicity may be attributable to the transforming capacity mediated by several viral oncogenes. The proliferative effect of E1A, the anti-apoptotic influence of E1B and E4orf6 proteins and the specific transforming activity of E4orf1 from type 9 have been extensively studied over the past decades [19], [20], [21], [22]. E1A and E1B proteins seem to be sufficient for oncogenic transformation of a number of rodent cells [21], [23] but also of some human cells [24], [25]. Additionally, E4orf6 has been shown to support the transformation potential in rodent cells remarkably [26], [27]. To date especially the oncogenic potential of the human HAdV type 12 and the species C type 5 are well studied in animal models.

In view of the well-documented ability of HAdV to induce sarcomas in rodents, we have used broad-spectrum PCR-based detection assays and FISH analyses to screen human sarcomas, including gastro-intestinal stroma tumors, leiomyosarcomas and liposarcomas, for the presence of HAdV sequences. In this study, we could detect, for the first time, the presence of HAdV inside infiltrating T-lymphocytes in different sarcoma tissue.

## Materials and Methods

### Patient samples and cells

In total 81 sarcoma specimens were investigated in this study, which were all archived paraffin-embedded tissue samples and written informed consent had been obtained. All samples were investigated anonymously. We have received a formal written waiver for the need of ethics approval from our institutional review board; Ethik Kommission der Arztekammer – Hamburg. The specimens include 22 gastro-intestinal stroma tumors (GIST) from the stomach, 30 leiomyosarcomas derived from soft muscle tissue of the uterus and 29 liposarcomas from various adipose tissues. All patient samples were provided by the Department of Pathology, University Medical Center Hamburg-Eppendorf. The cell lines HEK293 and A549 were obtained from the Leibniz Institute DSMZ-German Collection of Microorganisms and Cell Cultures. A549 cells were infected with HAdV type 5 (H5*pg*4100).

### DNA extraction

Three 10 µm-thick histological sections were derived from individual paraffin-embedded specimens. DNA extraction was carried out by the QIAmp Tissue DNA Mini Kit (QIAGEN, Germany) according to the manufacturer's recommendations [6] except protein K lysis was performed over night at 56°C.

### Real-time PCR analysis

A broad-spectrum HAdV qRT-PCR assay was used for the screening of the DNA samples as previously described [28]. The PCR tests were carried out using the Rotor Gene Q cycler (QIAGEN, Germany) and the TaqMan® Universal PCR Master Mix (Invitrogen). Approximately 100 ng DNA were used as template in individual PCR reactions. To control for adequate quantity and quality of the DNA templates, a single-copy human house-keeping gene (beta-2-microglobulin) was tested in parallel by qRT-PCR, as described [29], and samples were regarded as adequate for virus analysis if a minimum of 1×10^3^ copies of the control gene were present. For species identification in samples that tested positive by the broad-spectrum HAdV assay, a qRT-PCR to detect hexon and fiber genes was used [30]. In addition to the broad-spectrum HAdV qRT-PCR the detection of the adenoviral oncogene E1A was performed by a specific qRT-PCR assay using primers and a dual labeled probe as indicated in Table 1 for all sarcoma samples. HAdV reference strains obtained from the American-Type-Culture-Collection (ATCC) served as positive controls, and several negative controls were included in each assay. In order to exclude contamination of our tumor samples we additionally performed a qRT-PCR detecting the 1,8 kbp deleted laboratory adenovirus strain we use in our institute for studying in-vitro infections (Table 2). Precautions to avoid the possibility of contaminations have been taken like extraction of the DNA in rooms, where no plasmids with adenoviral DNA or infectious virus have been in. Moreover, all PCRs performed have been carried out in a UV decontaminated PCR work station. The statistic evaluation about the significance of adenovirus positivity in the total cohort of investigated specimens had been calculated by the two-sided t-test.

**Table 1:** Oligonucleotides for quantification and detection of HAdV.

Primer	Oligonucleotide sequence (5′–3′)
TaqMan assays^a^	
AdVC E1A for	GACGGCCCCCGAAGATC
AdVC E1A probe	CGAGGAGGCGGTTTCGCAGA
AdVC E1A rev	TCCTGCACCGCCAACATT
AdV5 E3del for	CTACGGGCTATTCTAATTCAGGTTTC
AdV5 E3del probe	TGCGCTGTTGCTCGGCCG
AdV5 E3del rev	CCATGTCTTGGAGCTCTTGATTC
Nested PCR for HAdV typing^b^	
AdVC E1 for	ATACCCGGTGAGTTCCTCAAG
AdVC E1 rev	CGCCATTTTAGGACGGCGG
AdVC E1 for n	ACTCTTGAGTGCCAGCG
AdVC E1 rev n	GGACGGCGGGTAGGTC

a The probe sequences indicated are TagMan probes labeled with 6-carboxyfluorescein on the 5′ end and with 6′-corboxytetramethylrhodamine on the 3′ end; ^b^ AdVC E1 for n and AdVC E1 rev n are primers for the second round of the nested PCR.

**Table 2:** qRT-PCR and sequencing analysis of adenovirus DNA in human sarcomas.

HAdV positive Sarcoma Sample ID	Hexon (P<0,01)	E1A (P<0,01)	species/type	HAdV5 E3del
GIST (1/19)				
18	-	positive	C	-
Leiomyosarcoma (6/22)				
32	positive	positive	C05	positive
34	-	positive	C05	-
38	positive	positive	C05	-
42	positive	positive	C05	positive
46	-	positive	C05	-
51	positive	positive	C05	-
Liposarcoma (8/23)				
61	positive	positive	C	-
62	positive	positive	C02/06	-
64	positive	positive	C05	-
65	positive	positive	C05	-
68	positive	positive	C05	-
70	-	positive	C05	-
71	-	positive	C05	-
80	positive	positive	B	-

All PCR positive sarcoma specimens are shown. In samples 32 and 42 the laboratory strain with a deletion in the E3 region was detected. In sample 62 a sequence identical for type 2 and type 6 has been identified. GIST: gastro intestinal stroma tumors.

### Nested PCR and sequencing analysis for HAdV-type identification

Primers for HAdV species-specific PCR were placed to amplify the E1 gene sequence. A nested PCR protocol was employed to generate sufficient amounts of DNA for type analysis in samples with low HAdV copy number. The first-round PCR reactions were carried out in 25 µl reactions containing 1 Unit of Fermentas DreamTaq DNA Polymerase (Fermentas, Germany), 3 mM MgCl_2_, 200 µM of each dNTP, 200 nM of primers (Metabion, Germany) and 5 µl template DNA extracted as described above. The amplification included initial denaturation at 95°C for 2 min, followed by 37 cycles with denaturation at 95°C for 30 sec, annealing at 58°C for 60 sec, and extension at 72°C for 30 sec, with a final elongation step at 68°C for 7 min. For the nested PCR different primers were used as listed in Table 1. The nested PCR reactions were carried out in 50 µl reactions containing the same reagents and concentrations as in the first round except for specific primers and 3 µl of the first reaction PCR product as template and an annealing temperature of 52°C. Subsequently the amplified fragment of the nested PCR was purified by the GeneJet Gel extraction Kit (Fermentas, Germany) and subjected to direct sequencing. Sequencing of the purified PCR products was performed in both directions with the sense and antisense primers used for the nested PCR by Seqlab (Seqlab sequencing laboratories, Germany). Sequences were analysed by standard nucleotide BLAST at the NCBI database and the multiple alignment was performed with the CLC Main Workbench 5 software.

### Fluorescence-in-situ-hybridization (FISH)

The specific adenovirus probe for hybridization was generated from a bacmid containing the whole adenovirus type 5 genome [31]. A second probe against the centromer 12 was kindly provided by Margit König (St. Anna Kinderkrebsforschung, Vienna, Austria). The probes were labeled by Nick-translation with biotin-11-dUTP or digoxigenin-12-dUTP (Roche, Germany) according to the manufacturers' recommendations. The hybridization protocol is summarized as follows: 5 µm thickness tissue sections from paraffin-embedded tissue were placed onto coated slides, heated at 70°C for 15 min and deparaffinized in Xylol. Proteolytic pretreatment was performed with pepsin (2,5 mg/ml) at 37°C. The slides were denatured at 85°C for 10 min and hybridization was carried out over night and subsequently washed in 0,4x sodium-saline citrate (SSC) at 72°C. Immunodetection with anti-FITC and anti-Biotin antibodies was performed as previously described [32]. Slides were washed in 4× SSC/0,6% Tween20 and mounted with a medium containing 5% glycerol in 10% polyvinylalcohol added to 25 mM Tris/HCl (pH8) and 2,5% DABCO. For the co-staining of adenovirus DNA and Bcl11b transcription factor on the same tissue sections, the FISH protocol was performed first as described above. Then Bcl11b staining was applied as previously described [33] using a rat-anti-Bcl11b antibody (Biolegend Inc) and a FITC anti-rat as secondary antibody. The hybridization was visualized for each tissue specimen using a Leica DMI 6000B fluorescence microscope equipped with appropriate filter sets. Images were taken with a charge-coupled device camera using the LAS AF software. Adobe Systems Photoshop 6.0 and Illustrator software were used to crop the images and design the illustrations. Additional hematoxylin-eosin (HE) stained sections were used for histological analysis of HAdV positive tumor tissues, both to define representative tumor regions and distinguish different cell types.

## Results

### Detection of human adenovirus in sarcoma tissue by qRT-PCR screening

We investigated the presence of human adenovirus DNA in sarcoma specimens from 13 patients. In total specimens from 81 sarcoma patients have been collected and the extracted DNA quality was assessed by PCR amplification of the human housekeeping gene, beta-2 microglobulin (B2M). The threshold to discard samples with poor or insufficient DNA quality was set at 5×10^3^ copies of the B2M gene. 17 out of the 81 samples were not used for further analysis due to low DNA quality. The other 64 samples included 19 GIST, 22 leiomyosarcomas and 23 liposarcomas.

**Figure 1:**
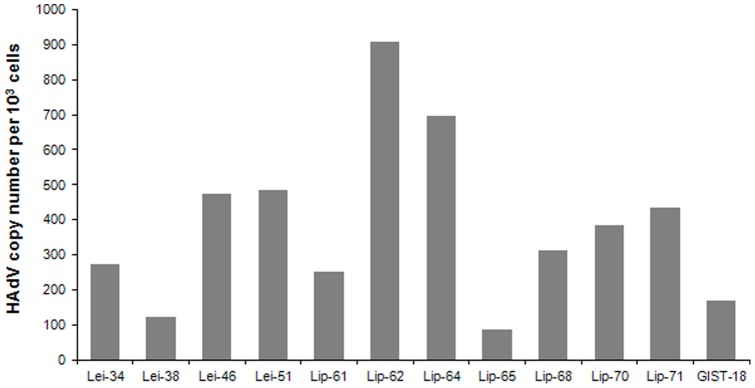
HAdV copy numbers in human sarcomas detected by qRT-PCR. qRT-PCR was performed for the hexon gene of HAdV and for beta-2-microglobulin, B2M (a single human house-keeping gene). The calculation of the viral copies is related to the copy number of the house-keeping gene for each specimen. The virus copy numbers per 10^3^ cells are shown for 4 leiomyosarcoma (Lei), 7 liposarcoma (Lip) and one GIST (gastro intestinal stroma tumor).

Screening with the Pan-AdV rRT-PCR, which targets the hexon (coding for the major capsid protein) showed the presence of HAdV in 4 leiomyosarcomas and 6 liposarcomas (P<0,01). Additionally, we performed a screening to detect the E1A gene in all 64 samples that resulted in a higher number of HAdV positive specimens including 1 GIST, 6 leiomyosarcomas and 8 liposarcomas (P<0,01, Table 2). The number of viral genomes in each HAdV-positive tumor sample was investigated as previously described [29], finding a range of 88 to 907 viral copies per 10^3^ cells that could be detected in 12 HAdV-positive patient samples (Figure 1). When a species specific qRT-PCR was performed to distinguish between the different HAdV species, C types were found in 14 of 15 samples positive for HAdV. Only in one case HAdV species B could be detected. To exclude contamination of the tissue specimens, during DNA extraction and PCR, with HAdV laboratory strains (i.e. H5*pg*4100 [31]), we discriminated by qRT-PCR between the E3 deleted laboratory strains and the wild type HAdV from clinical isolates. This control PCR revealed two leiomyosarcomas containing a laboratory strain in sample 32 and sample 42, whereas the other 13 positive sarcomas showed the presence of wild type HAdV (Table 2).

### HAdV type 5 occurs most frequently in human sarcoma specimens

**Figure 2:**
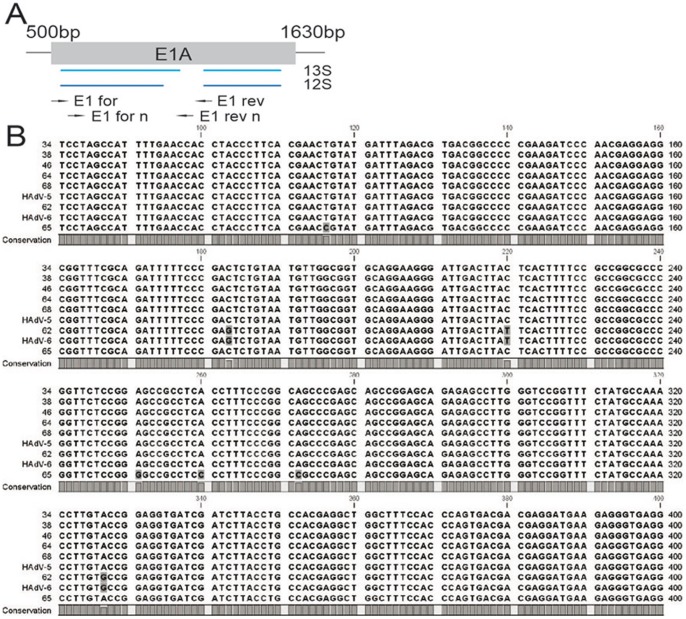
Typing of adenovirus strains present in human sarcoma specimens. E1A has been amplified by nested PCR in species C positive samples. A) A detailed scheme shows the binding sites of the oligonucleotides for the first and second round of the nested PCR. B) 531bps of the E1A gene have been sequenced from 7 different samples. A part of the sequences investigated are depicted in an alignment with two reference GenBank sequences of HAdV-5 (NCBI acc. no.: AY339865) and HAdV-6 (NCBI acc. no.: HQ413315.1).

An adequate DNA amount and quality for HAdV type identification was in most but not in all HAdV-positive sarcoma specimens available. In order to determine which HAdV types were present in the different virus-positive sarcoma samples, we performed a nested-PCR, amplifying parts of the E1 region (Figure 2A). Subsequent BLAST analysis of the PCR products through the NCBI database revealed that in 9 out of 13 samples E1A sequences were homologous to HAdV-5. From those 8 samples showed complete homology of the E1A sequence with the HAdV-5 strain with the NCBI accession number AY339865.1, whereas only one sample (number 65) presented a HAdV-5 strain that has never been described before. Interestingly, sample 62 showed homology to the E1A sequence of both HAdV-2 and HAdV-6, also from species C (NCBI accession no. HQ413315.1, HQ003817.1, FJ349096.1 and JO1917.1) (Figure 2B). Due to complete homology of the E1A gene in these strains we could not discriminate between types 2 and 6. Additionally, the individual HAdV type in two positive species C sarcomas (sample 18 and 61) and in one species B positive sarcoma (sample 80) could not be identified.

### Persistent adenoviral DNA in leiomyo- and liposarcoma tissue sections

**Figure 3:**
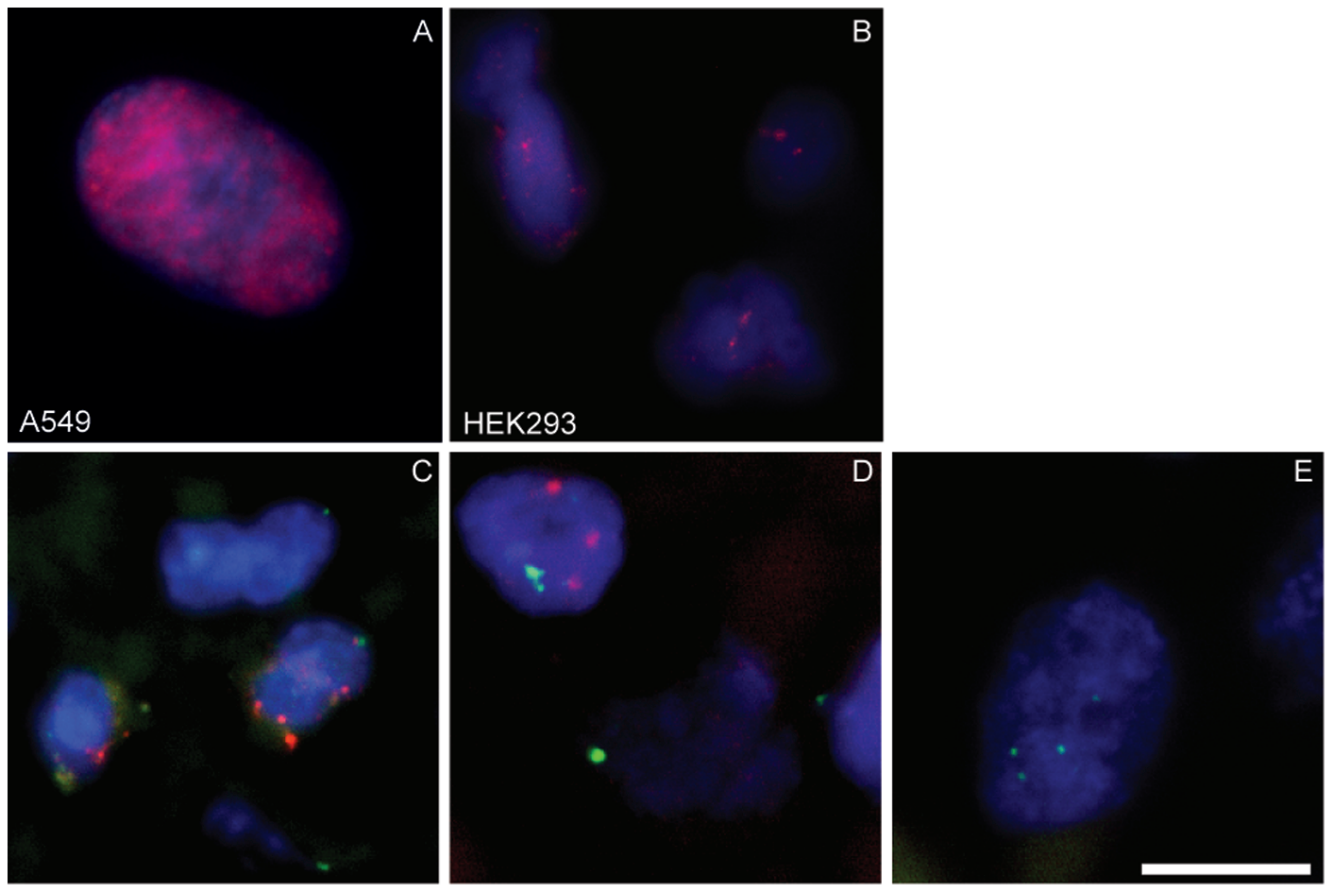
Detection of HAdV by Fluorescence-in-situ-hybridization (FISH). The hybridization was performed in all experiments with a biotinylated adenovirus DNA probe. Control hybridization was performed on (A) A549 cells infected with HAdV type 5 (24 h, MOI 50) and (B) HEK293. Paraffin-embedded human sarcoma tissue sections were additionally hybridized for the internal positive control (DIG labelled centromeric probe q12). FISH results from (C) a PCR positive leiomyosarcoma (sample 51), (D) a liposarcoma (sample 70) and (E) a PCR negative liposarcoma (sample 47) are shown. For the detection of HAdV-DNA the mouse anti-Biotin-Cy3 antibody (Jackson ImmunoResearch) and for the centromeric probe the sheep-anti-digoxigenin-FITC labelled antibody (Roche) have been used. The bar in picture E represents 10 µm.

In order to investigate the distribution of the HAdV DNA in virus positive sarcoma samples we carried out a fluorescence in situ hybridization (FISH) in several tissue sections. In these experiments we included two different controls to compare with: The first one was *in vitro* infected A549 cells, which reflects a lytic viral life cycle due to the intensive production of viral progeny. The staining of these cells showed characteristic viral replication centres in the nucleus detected by a Cy3 antibody against the HAdV-probe (Figure 3A). The second control sample was a staining of HEK293 cells, which carry integrated parts of the adenoviral genome and showed a different FISH picture with two to three distinct HAdV-signals in each cell (Figure 3B). Hybridization of paraffin-embedded sarcoma tissue sections with the HAdV-probe resulted in 3 positive FISH-stained out of 12 PCR positive samples (samples 51, 70 and 71) with specific signals (Figures 3C and 3D). In concordance with the PCR results sample 47 revealed no HAdV-presence by FISH analysis (Figure 3E). Interestingly, the distinct signals in the tumor sections showed a more similar pattern to those detected in HEK293 cells, than to the *in-vitro* infected A549 cells. A DIG-labelled centromeric probe served as internal positive control and was detected by an anti-FITC antibody. The FITC signals in nearly all cells provide evidence for reliable hybridization on paraffin-embedded tissues (Figure 3E–3E).

### HAdV DNA specifically localizes in infiltrating T-lymphocytes inside the sarcoma tissue

**Figure 4:**
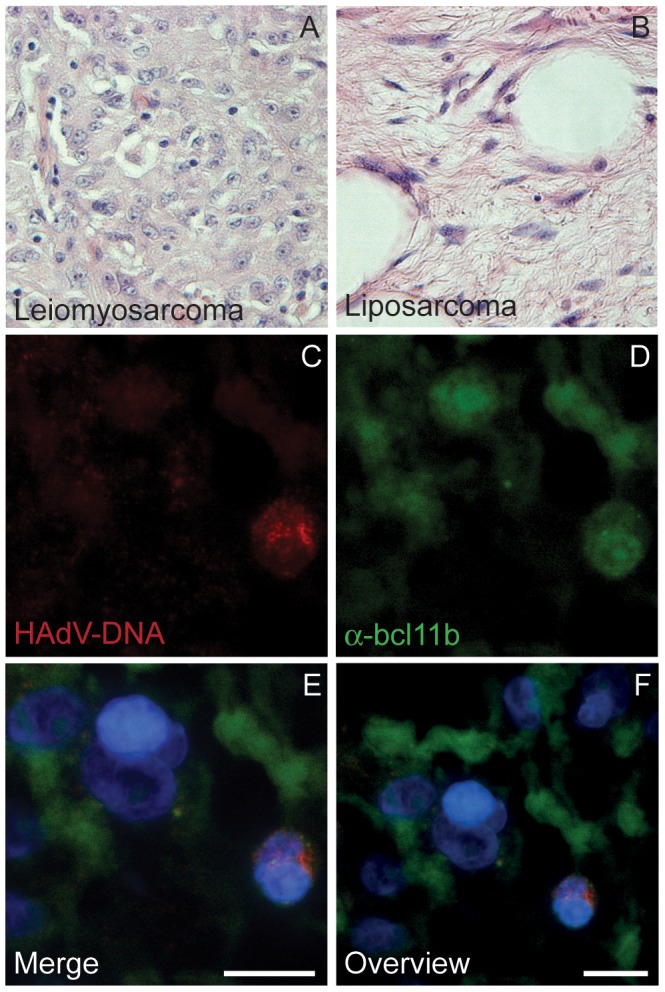
Histology of leiomyo- and liposarcoma and lymphocyte staining. HE-staining of paraffin embedded tissue sections from (A) leiomyosarcoma and (B) liposarcoma are shown (400-fold magnification). FISH-analysis for HAdV DNA (C) and co-staining for the transcription factor Bcl11b (D) has been performed on liposarcoma tissue sections. (E, F) Merged pictures of the FISH (red), bclb11 (green) and DAPI staining at different sizes are displayed. The bars in picture E and F represent 10 µm.

Although HAdV DNA could be detected in both leiomyo- and liposarcomas, not every cell was positive for FISH staining but several scattered positive cells were distributed over the tumor tissue sections. This distribution of HAdV DNA in partly infected cells has been already shown by FISH analysis in tonsillar T-lymphocytes (unpublished data), which have been shown to bear persistent HAdV infections [4]. To further characterize the specific cell type where HAdV was found in the samples assayed, we first analysed Hematoxilin-Eosin (HE) stained adenovirus positive sample sections (Figures 4A and 4B, respectively). Liposarcoma samples showed characteristic vacuoles of fatty cells, whereas leiomyosarcoma displayed a more homogeneous picture and hematopoietic cells could be easily distinguished evenly distributed in all tissue sections. Human sarcoma tissue sections with positive FISH signals have been evaluated by pathologic experts and the cells containing HAdV DNA positive signals were found in all cases in the interstitium, where also blood components are interspersed. In figures 4E and 4F tumor cells can be seen beside cells with a smaller and round shaped morphology. These smaller cells show also a more intense DAPI staining. The FISH signals have been exclusively found in these smaller cells (Figure 4C) and co-staining of the tissue sections for the transcription factor Bcl11b was also found in these cells (Figure 4D). This protein is a transcription factor that is expressed in all kind of T-lymphocytes [34]. In figure 4D and in the merged pictures (4E and 4F) the signals for Bcl11b can be found only in small cells with intense DAPI staining but not in the leiomyo- or liposacorma tumor cells. As shown in Figures 4C–E, only those smaller and rounded cells showed co-localization of both FISH and Bcl11b, indicating that HAdV DNA is specifically found in the infiltrated T-lymphocytes inside sarcoma tissue samples.

## Discussion

Persistence of HAdV is well described to occur in T-lymphocytes from tonsils/adenoids and intestines [4], [7]. Moreover, there are data about the presence of the virus in liver, lung epithelium and small-cell lung cancers and brain cells [6], [18], [35], [36]. The occurrence of HAdV in T-lymphocytes has not been correlated with any malignancy since screening of T-cell associated tumors did not result in a significant finding of the virus so far [6], [37]. In lung and brain tumors HAdV was frequently present in the investigated samples. However a correlation between viral infection and tumor initiation has not been proven yet. Moreover, HAdVs are well known to induce sarcomas in rodents since decades [23]. For these reasons, we investigated the presence of HAdV in different types of human sarcomas. By using a specific qRT-PCR assay we screened a subset of 64 human sarcoma specimens and detected HAdV E1A and/or hexon in 35% of the tested liposarcoma and in 18% of the leiomyosarcoma specimens. Interestingly, although the amount of infected cells was rather low, viral DNA was specifically found in the T-lymphocytes infiltrated in between the tumor cells, supporting the notion that lymphocytes are a common site of adenovirus persistence.

Among human neoplasms, soft tissue sarcomas are relatively rare, representing about 1% [38]. The liposarcoma is a tumor derived from cells that undergo adipose differentiation and ranges from lesions that are essentially benign to those that are malignant, more aggressive and likely to metastasize. On the other hand, all leiomyosarcoma are aggressive tumors that are difficult to treat. They derive from smooth muscle cells typically of uterine, gastrointestinal or soft tissue origin. The prognosis is poor, with survival rates among the lowest of all soft tissue sarcomas [39], [40]. Both sarcomas are well vascularized and we could find lymphocytes to be spread in the tissue sections. We did not find any HAdV FISH signal in the tumor cells we investigated. For this reason we cannot assume a correlation of the tumor with the adenovirus infection. However, the finding of the virus in lymphocytes seems to be of interest since HAdV has already been detected in T-cells of tonsils, adenoids and the intestine. In addition the similar observation of rare cells with distinct FISH signals, like it was already seen in tonsillar lymphocytes support the idea of persistent adenovirus DNA in lymphocytes. The occurrence of the virus in infiltrated lymphocytes in the malignant tissue seems to be also of interest due to a possible reactivation in the immunosuppressed host. In tonsils and adenoids the exclusive presence of species C types has been described, whereas in the intestine especially type 4 from species E has been found [41], [42], [43]. Sequence analyses revealed also a high prevalence of C types in our specimens, mainly type 5. To date frequent findings of species C types have been described for immunosuppressed patients [44], [45], in a persistent state in children [4] and as a cause of respiratory tract infections occurring in the early childhood [46]. The preferential persistence of species C types in T-lymphocytes might be because of the absent lytic infectious cycle. The repression of virus production in T-cells seems to be inhibited already at the stage of viral replication [47]. The absence of the coxsackie adenovirus receptor (CAR) in these cells might also have relevance for this fact [48]. However, a smaller amount of viral particles seems to be able to enter the cells via a receptor independent way through integrins [49]. In this context additional, so far unknown, mechanisms could be a cause for the persistent status of HAdV in T-cells.

In our study we analysed the presence of viral DNA but we have no evidence of viral proteins in the tumor tissue. Since protein detection is difficult in paraffin material, the PCR and FISH techniques we applied are even more sensitive for the detection of adenoviral persistence than immunohistochemical methods on tissue sections. Considering the possibility that immunohistochemistry might provide false negative results we did no protein analysis with the paraffin embedded tissue. However, it would be of interest if the viral proteins could be found in fresh or frozen sarcoma specimens or a reactivation of the persistent status can be triggered.

The finding of the HAdV DNA in the infiltrating lymphocytes gives evidence for a persistence of this virus in lymphocytes of sarcomas from fatty tissue. A correlation with the investigated tumor entities cannot be given but the persistence in lymphocytes seems to be tissue-dependent since in tumors located in the stomach (GIST), and also a number of other tumors screened showed complete absence of the virus [6], [17], [50]. Previous reports revealed also a correlation of HAdV type 36 infections and obesity [51], [52]. Maybe fatty tissue is kind of a reservoir for persistently infected cells like it is described for the occurrence of HAdV in liver [36]. Reactivation of the virus is suggested to be involved in the outbreak of severe adenoviral infection in patients undergoing allogeneic stem cell transplantation [44]. If a reactivation of persistent HAdV in lymphocytes is involved in the outbreak of different diseases and might has also an effect on malignant tissue needs additional investigation.
